# Nonregistration, Discontinuation, and Nonpublication of Randomized Trials: A Systematic Review

**DOI:** 10.1001/jamanetworkopen.2025.24440

**Published:** 2025-09-03

**Authors:** Benjamin Speich, Ala Taji Heravi, Christof M. Schönenberger, Lena Hausheer, Dmitry Gryaznov, Jason W. Busse, Manuela Covino, Szimonetta Lohner, Malena Chiaborelli, Johannes M. Schwenke, Ruben Ramirez Zegarra, Ramon Saccilotto, Julia M. Hüllstrung, Erik von Elm, Arnav Agarwal, Julian Hirt, David Mall, Alain Amstutz, Selina Epp, Dominik Mertz, Anette Blümle, Belinda von Niederhäusern, Ayodele Odutayo, Alexandra N. Griessbach, Sally Hopewell, Matthias Briel

**Affiliations:** 1 Division of Clinical Epidemiology, Department of Clinical Research, University Hospital Basel, University of Basel, Basel, Switzerland; 2 Department of Anesthesia, McMaster University, Hamilton, Ontario, Canada; 3 Department of Health Research Methods, Evidence, and Impact, McMaster University, Hamilton, Canada; 4 MTA–PTE Lendület “Momentum” Evidence in Medicine Research Group, Department of Public Health Medicine, Medical School, University of Pécs, Pécs, Hungary; 5 Department of Medicine and Surgery, Obstetrics and Gynecology Unit, University of Parma, Parma, Italy; 6 Clinical Trial Unit, Department of Clinical Research, University of Basel and University Hospital Basel, Basel, Switzerland; 7 Cochrane Switzerland, c/o Cochrane Germany Foundation, Freiburg, Germany; 8 Division of General Internal Medicine, Department of Medicine, University of Alberta, Edmonton, Alberta, Canada; 9 Research Center for Clinical Neuroimmunology and Neuroscience Basel (RC2NB), University Hospital Basel and University of Basel, Basel, Switzerland; 10 Population Health Sciences, Bristol Medical School, University of Bristol, UK; 11 Oslo Centre for Biostatistics and Epidemiology, Oslo University Hospital, University of Oslo, Norway; 12 Department of Medicine, McMaster University, Hamilton, Ontario, Canada; 13 Clinical Trials Unit, Medical Center – University of Freiburg, Faculty of Medicine, University of Freiburg, Germany; 14 Roche Pharma AG, Grenzach-Wyhlen, Germany; 15 University Health Network, Division of Nephrology, Department of Medicine, University of Toronto, Ontario, Canada; 16 Centre for Statistics in Medicine, Nuffield Department of Orthopaedics, Rheumatology and Musculoskeletal Sciences, University of Oxford, Oxford, UK

## Abstract

**Importance:**

Previous work found that 25% to 30% of randomized clinical trials (RCTs) with protocols approved in 2012 or between 2000 and 2003 were discontinued prematurely, most commonly due to inadequate participant recruitment. To minimize research waste, RCTs should be registered and their results made available.

**Objectives:**

To assess the fate of RCTs approved by ethics committees in 2016 in terms of nonregistration, discontinuation, and nonpublication, and to examine RCT characteristics associated with discontinuation due to poor recruitment and nonpublication of RCT results.

**Evidence Review:**

As a prespecified project of the Adherence to SPIRIT Recommendations (ASPIRE) study, this systematic review had access to 347 RCT protocols approved in 2016 by research ethics committees in the UK, Switzerland, Germany, and Canada. Eligible RCTs were defined as prospective studies randomly assigning participants to interventions to study effects on health outcomes. RCTs were excluded that never started, were ongoing at time of follow-up, were duplicates, or were labeled as pilot, feasibility, or phase 1 trials. Key trial characteristics were extracted from the approved trial protocols. In July 2024, pairs of reviewers systematically searched for trial registrations and results publications. When the status of either was unclear, the corresponding ethics committee or the principal investigator was contacted for clarification.

**Findings:**

Of 347 included RCTs, 20 (5.8%) were unregistered, 108 (31.1%) were discontinued, most often due to poor recruitment (49 [45.4%]), and 276 (79.5%) made their results publicly available. Results from industry-sponsored trials were more often available than non–industry-sponsored trials (166 of 181 [92.3%] vs 110 of 166 [66.3%]). This difference was attributable to a higher prevalence of industry-sponsored trials that reported results in trial registries (153 of 181 [84.5%]) vs nonindustry RCTs (17 of 166 [10.2%]). Multivariable logistic regression indicated that industry-sponsored trials were less frequently discontinued due to poor recruitment than non–industry-sponsored RCTs (adjusted odds ratio, 0.32 [95% CI, 0.15-0.71]).

**Conclusions and Relevance:**

Findings from this systematic review indicated that nonregistration, premature discontinuation due to poor recruitment, and nonpublication of RCT results remained major challenges, especially for non–industry-sponsored trials. To mitigate these challenges, requirements enforced by funders and ethics committees also taking into account legal obligations should be considered and empirically evaluated.

## Key Points

**Question:**

What percentage of randomized clinical trials (RCTs) are unregistered, prematurely discontinued, or unpublished?

**Findings:**

This systematic review assessing 347 RCTs receiving ethical approval in 2016 in the UK, Switzerland, Germany, and Canada found that 5.8% were unregistered, 31.1% were discontinued (predominately because of poor recruitment), and 20.5% did not make results available. Industry-sponsored RCTs performed better than non–industry-sponsored RCTs in making trial results available (often through trial registries) and industry-sponsored RCTs were less likely to be discontinued due to poor recruitment.

**Meaning:**

Results of this study suggest that actions are needed to improve good practice and transparency in RCTs, particularly among non–industry-sponsored trials.

## Teaser

This systematic review describes the percentages of randomized clinical trials approved in 2016 that are unregistered, prematurely discontinued, or unpublished.

## Introduction

Randomized clinical trials (RCTs) are critical for evaluating clinical interventions,^1,2^ and nonpublication of RCT findings may compromise the available evidence by distorting the apparent safety and efficacy data of interventions.^3^ To mitigate publication bias, clinical trial registries were introduced, and prospective registration of RCTs (ie, before the enrollment of the first participant) in a clinical trial registry became mandatory.^4^ Despite this requirement, the publication of unregistered or retrospectively registered RCTs is frequently observed.^5,6^ Another challenge is premature discontinuation of RCTs, typically due to recruitment that is slower than expected, leading to considerable research waste as the primary research question cannot be answered conclusively.^7^ Furthermore, results from discontinued RCTs are less frequently published than those of completed RCTs, although their results can provide valuable evidence for decision-making through meta-analyses.^8^

A previous study^8^ assessing RCT protocols that were approved by research ethics committees (RECs) in Switzerland, Germany, and Canada between 2000 and 2003 found that 25% of RCTs were discontinued (primarily due to poor recruitment), and that only 59% made their results publicly available. Repeating this study (also including RECs from the UK) with protocols approved in 2012, members of our team found that 30% of RCTs were discontinued, but the percentage of trials that made their results available had increased to 87%.^9^ Compared with industry-sponsored trials, nonindustry trials were less likely to provide results; 10% were unregistered (vs 2% for industry-sponsored), and only 16% published results on trial registries (vs 84% for industry-sponsored RCTs).^9^ In this updated systematic review assessing RCT protocols approved in 2016, we assessed (1) the percentage of unregistered RCTs, (2) the percentage of discontinued RCTs and the reasons for discontinuation, (3) the public availability of the RCT results, and (4) the percentage of trials sharing results in trial registries. We investigated trial characteristics associated with nonpublication of trial results and trial discontinuation due to poor recruitment. In addition, we compared these findings to previous studies from members of our team assessing RCTs approved in 2012^9^ and between 2000 and 2003.^8^

## Methods

This systematic review was conducted in collaboration with the following ethical committees, which granted access to ethically approved RCT protocols: all 7 Swiss REC committees,^10^ the Bristol office of the UK National Research Ethics Service, which oversees 19 RECs across the UK, the University of Freiburg Medical Centre Ethical Committee in Germany, and the Hamilton Integrated Research Ethics Board in Canada. We reported this systematic review adhering to the Preferred Reporting Items for Systematic Reviews and Meta-Analyses (PRISMA) reporting guideline.^11^ This work was conducted as part of a PhD thesis (A.T.H.).^12^

### Study Sample

This study was a prespecified project of the Adherence to SPIRIT Recommendations (ASPIRE) study,^13^ which assessed the reporting quality of REC-approved RCT protocols before and after the publication of the Standard Protocol Items: Recommendations for Interventional Trials (SPIRIT) reporting guideline (published in 2013).^14,15^ For the ASPIRE project, we obtained access to RCT protocols that were approved by RECs in 2012 and 2016 in Switzerland, the UK, Canada, and Germany. Eligible RCTs were defined as prospective studies assigning participants randomly to different interventions to study effects on health outcomes. RCTs were excluded that never started (ie, did not recruit any participant), were still ongoing at time of follow-up, were duplicates, or were labeled as pilot, feasibility, or phase 1 trials.

### Data Collection

Within the ASPIRE study, members of our team extracted key characteristics from approved trial protocols (ie, type of sponsorship, population, intervention, control, primary outcome, planned sample size, and trial registration number), and assessed adherence to the SPIRIT reporting guideline (results published separately^16,17^). For the current study, using information from trial protocols, pairs of reviewers systematically searched for evidence of trial registration and for publication of trial results approximately 8.5 years after receiving ethical approval (last search conducted in July 2024). For registration status, we checked whether the registration numbers provided in the protocol were correct. If no registration number was provided, we searched for trial registration in the World Health Organization International Clinical Trial Registry Platform database, the US National Library of Medicine (ClinicalTrials.gov), the European Union Clinical Trial Registry, and the International Standard Randomized Controlled Trial Number registry. Finally we used the Google search engine if no information on registration was identified. RCTs were classified as unregistered if we could not find a registration with this search strategy. For trial publication status, we searched PubMed, Google Scholar and Scopus for potential full text publications. We looked for registration and publication by searching for full titles, short titles, study acronyms, and the study population and intervention (with or without specifying the control group or name of the investigator, if available).

We extracted the date of trial registration, date of first participant enrollment, trial status (ie, completed, premature discontinuation together with reason, or unclear), availability of trial results in a registry (results directly published in registry), and the planned and achieved sample sizes. RCTs that reported their status as ongoing but were not updated within the last 2 years were classified as unclear (only for trials with an estimated completion date before July 2024). RCTs were classified as discontinued if they were specifically labeled as such by investigators or if they recruited less than 90% of the planned sample size prespecified in the approved study protocol. We contacted RECs and 89 trial investigators by email and phone (using up to 3 reminders or approaches; last contact in October 2024; 61 responses received) to resolve trial status if unclear or to ascertain the reason for trial discontinuation or for those that lacked publicly available trial results.

### Statistical Analysis

We calculated the percentage of trials that were registered, completed, and provided publicly available results, with corresponding 95% CIs. For availability of trial results, we included sharing results in trial registries or through a peer-reviewed publication. Among registered trials, we assessed the percentage that were registered retrospectively (ie, the first patient was recruited before the trial was registered). All analyses were stratified by type of sponsor (industry vs nonindustry) and country where ethics approval was received. We descriptively compared our results for RCT protocols approved in 2016 with findings of prior studies of protocols by members of our team approved in 2012^9^ and the early 2000s.^8^ To ensure a fair comparison, we added an additional analysis assessing trial results availability, in which we limited the follow-up duration of the RCTs approved in 2012 to 8.5 years—the same follow-up duration as for RCTs approved in 2016.

We constructed univariable and multivariable logistic regression models to assess whether better adherence to reporting guidelines in the protocol, larger target sample size, use of an active comparator vs placebo, multicenter vs single-center trial, reporting of any recruitment projection vs not reporting, industry-sponsored vs investigator-sponsored trials were associated with public availability of trial results, or premature trial discontinuation due to poor recruitment. Details on additional conducted sensitivity analyses, including data from RCTs approved in 2012,^9^ and accounting for potential cluster effects are presented in the eMethods in Supplement 1. Analyses were conducted in July 2024 using Stata, version 16.1 (StataCorp LLC), with a 2-sided α of .05 as the threshold for statistical significance.

## Results

We included 347 RCT protocols that were approved in 2016 by RECs in Switzerland, Germany, Canada, or the UK (Figure). Included RCTs had a median planned sample size of 220 (IQR, 102-450) participants and adequately reported a median of 76% (IQR, 68%-81%) SPIRIT items (Table 1). Most of 347 RCTs used a parallel arm design (322 [92.8%]), were multicenter (265 [76.4%]), and tested drugs (212 [61.1%]). Approximately half of trials (181 [52.2%]) were industry sponsored. Further information on baseline characteristics is presented in Table 1, eTable 1 in Supplement 1 (stratification by country of the REC providing approval), and eTable 2 in Supplement 1 (baseline characteristics of trial protocols receiving ethical approval in 2000-2003,^8^ 2012,^9^ and 2016).

**Figure.**
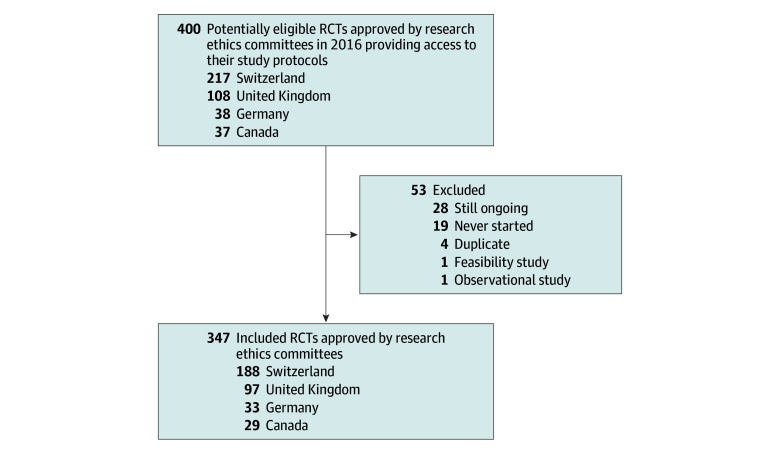
Flowchart of Included Randomized Clinical Trials (RCTs)

**Table 1.** Characteristics of Included RCTs

Characteristic	RCT, No. (%)		
	Industry sponsored (n = 181)	Nonindustry sponsored (n = 166)	All (N = 347)
Planned sample size, median (IQR)	300 (140-555)	150 (80-344)	220 (102-450)
Proportion of adequately reported SPIRIT items in protocol, median (IQR)	0.77 (0.71-0.81)	0.75 (0.62-0.81)	0.76 (0.68-0.81)
Single center	5 (3.3)	76 (45.8)	82 (23.6)
Multicenter	175 (96.7)	90 (54.2)	265 (76.4)
Study design			
Parallel	175 (96.7)	147 (88.6)	322 (92.8)
Crossover	2 (1.1)	11 (6.6)	13 (3.7)
Factorial	1 (0.6)	4 (2.4)	5 (1.4)
Cluster	1 (0.6)	3 (1.8)	4 (1.2)
Split body	2 (1.1)	1 (0.6)	3 (0.9)
Placebo-controlled	108 (59.7)	44 (26.5)	152 (43.8)
Recruitment rate reported in protocol	32 (17.7)	49 (29.5)	81 (23.3)
Country of ethical approval			
Switzerland	79 (43.6)	109 (65.7)	188 (54.2)
UK	60 (33.1)	37 (22.3)	97 (28.0)
Germany	26 (14.4)	7 (4.2)	33 (9.5)
Canada	16 (8.8)	13 (7.8)	29 (8.4)
Intervention			
Drug	153 (84.5)	59 (35.5)	212 (61.1)
Medical device	17 (9.4)	26 (15.7)	43 (12.4)
Behavioral	2 (1.1)	34 (20.5)	36 (10.4)
Surgical	2 (1.1)	22 (13.3)	24 (6.9)
Other^a^	7 (3.9)	25 (15.1)	32 (9.2)
Medical field			
Oncology	42 (23.2)	17 (10.2)	59 (17.0)
Cardiovascular	27 (14.9)	17 (10.2)	44 (12.7)
Surgical	7 (3.9)	24 (14.5)	31 (8.9)
Neurology	17 (9.4)	11 (6.6)	28 (8.1)
Psychiatry	0	27 (16.3)	27 (7.8)
Other^b^	88 (48.6)	70 (42.2)	158 (45.5)

Abbreviations: RCTs, randomized clinical trials; SPIRIT, Standard Protocol Items: Recommendations for Interventional Trials.

^a^ Process optimization in health care (n = 9); rehabilitation (n = 8); dietary supplement (n = 7); diagnostic (n = 3), creams and cosmetics (n = 3); vaccines (n = 2).

^b^ Gastrointestinal (n = 23); orthopedics and rheumatology (n = 22); respiratory (n = 20); pediatrics (n = 16); intensive care (n = 15); endocrinology (n = 13); hematology (n = 8); infectious diseases (n = 8); dentistry (n = 6); dermatology (n = 5); nephrology (n = 5); ophthalmology (n = 5); obstetrics and gynecology (n = 4); geriatrics (n = 4); hepatology (n = 1); public health (n = 1); ear (n = 1); primary care (n = 1).

Of 347 RCTs, 327 (94.2%) were registered (Table 2). Nonregistration and retrospective registration (ie, registration after the recruitment of the first patient) were more common among nonindustry sponsored trials (15 of 166 [9.0%] not registered; 24 of 166 [14.5%] retrospectively registered) compared with industry sponsored trials (5 of 181 [2.8%] not registered; 9 of 181 [5.0%] retrospectively registered). Results were available 8.5 years after receiving ethical approval (considering peer-reviewed publications and results in trial registries) for 276 of 347 (79.5%) RCTs. Industry-sponsored trials were more likely to have made results available in both peer-reviewed publications (140 of 181 [77.4%] industry-sponsored RCTs vs 109 of 166 [65.7%] non–industry-sponsored RCTs) and trial registries (153 of 181 [84.5%] industry-sponsored RCTs vs 17 of 166 [10.2%] nonindustry sponsored RCTs). Overall, results of industry-sponsored RCTs were publicly available in 166 of 181 trials (91.7%) and of non–industry-sponsored RCTs in 110 of 166 trials (66.3%). Trial results were less often available in registries among RCTs that received ethical approval in Switzerland (68 of 188 [36.2%]) compared with other countries (eTable 3 in Supplement 1). Of 98 RCTs that were not published in a peer-reviewed journal, 80 (81.6%) were registered and 27 (27.6%) made their results available in a registry (26 of 41 [63.4%] industry-sponsored RCTs vs 1 of 57 [1.8%] non–industry-sponsored RCTs) (Table 2).

**Table 2.** Nonregistration, Discontinuation, and Nonpublication of RCTs Receiving Ethical Approval in 2016

Variable	RCT, No. (%) [95% CI]		
	Industry sponsored (n = 181)	Nonindustry sponsored (n = 166)	All (N = 347)
Registration status			
Registered	176 (97.2) [93.7-99.1]	151 (91.0) [85.5-94.9]	327 (94.2) [91.2-96.4]
Prospectively registered	167 (92.3) [87.4-95.7]	127 (76.5) [69.3-82.7]	294 (84.7) [80.5-88.3]
Retrospectively registered	9 (5.0) [2.3-9.2]	24 (14.5) [9.5-20.7]	33 (9.5) [6.7-13.1]
Not registered	5 (2.8) [0.9-6.3]	15 (9.0) [5.1-14.5]	20 (5.8) [3.6-8.8]
Completion status			
Completed	125 (69.1) [61.8-75.7]	101 (60.8) [53.0-68.3]	226 (65.1) [59.9-70.1]
Discontinued	52 (28.7) [22.3-35.9]	56 (33.7) [26.6-41.5]	108 (31.1) [26.3-36.3]
Unclear	4 (2.2) [0.6-5.6]	9 (5.4) [2.5-10.0]	13 (3.7) [2.0-6.3]
Results availability			
At any source (peer-reviewed publication or in a trial registry)	166 (91.7) [86.7-95.3]	110 (66.3) [58.5-73.4]	276 (79.5) [74.9-83.7]
Peer-reviewed publication	140 (77.3) [70.6-83.2]	109 (65.7) [57.9-72.8]	249 (71.8) [66.7-76.4]
In trial registry	153 (84.5) [78.4-89.5]	17 (10.2) [6.1-15.9]	170 (49.0) [43.6-54.4]
Results not available (neither as publication nor in trial registry)	15 (8.3) [4.7-13.3]	56 (33.7) [26.6-41.5]	71 (20.5) [16.3-25.1]
Neither registered nor published	4 (2.2) [0.6-5.6]	14 (8.4) [4.7-13.7]	18 (5.2) [3.1-8.1]
Not published in peer-reviewed journal but registered^a^	37 (90.2) [76.9-97.3]	43 (75.4) [62.2-85.9]	80 (81.6) [72.5-88.7]
Not published in peer-reviewed journal but results available in registry^a^	26 (63.4) [46.9-77.9]	1 (1.8) [0.0-9.4]	27 (27.6) [19.0-37.5]

Abbreviation: RCTs, randomized clinical trials.

^a^ Only a subsample of 98 trials (41 industry-sponsored; 57 non–industry-sponsored) considered that were not published in a peer-reviewed journal.

Compared with trial protocols approved in 2012, the percentage of unregistered trials (5.9% among those approved in 2012 vs 5.8% approved in 2016) and retrospectively registered studies (10.1% among those approved in 2012 vs 9.5% approved in 2016) remained unchanged, with 1 of 4 non–industry-sponsored RCTs not prospectively registered (eTable 4 in Supplement 1). The availability of trial results increased substantially from trials with protocols approved in the early 2000s (59.3%) to those approved in 2012 (81.9% at the 8.5-year follow-up; 87.1% at the 10-year follow-up) (eTable 5 in Supplement 1) and RCTs approved in 2016 (79.5% at the 8.5-year follow-up). Among non–industry-sponsored RCTs, trial results were rarely made available in trial registries for protocols approved in 2012 (23 of 147 [15.7%]) or in 2016 (17 of 166 [10.2%]) (eTable 4 in Supplement 1).

In total, 108 of 347 (31.1%) of RCTs approved in 2016 were prematurely discontinued (Table 3). The most common reason for early discontinuation was poor recruitment (49 [45.4%]), followed by organizational and strategic reasons (13 [12.0%]) and futility (12 [11.2%]). Compared with completed RCTs, prematurely discontinued trials had higher odds of remaining unpublished, whether considering peer-reviewed publications only (odds ratio [OR], 4.09 [95% CI, 2.36-7.09]), reporting in trial registries only (OR, 1.94 [95% CI, 1.19-3.18]) (eTable 6 in Supplement 1) or either source (OR, 4.87 [95% CI, 2.07-7.27]). The percentage of discontinued trials remained consistent during the last 2 decades (27.9% in 2000-2003 vs 31.1% in 2106) and was most often attributed to poor recruitment (40.0% in 2000-2003 vs 45.4% in 2016) of prematurely discontinued trials (eTable 4 in Supplement 1).

**Table 3.** Reasons for Trial Discontinuation and Availability of Results

Reasons for discontinuation	RCTs, No. (%)						
	Discontinued			Availability status			
	All (n = 108)	Industry sponsored (n = 52)	Nonindustry sponsored (n = 56)	Any results (peer-reviewed or at trial registry)	As a peer-reviewed publication	In clinical trial register	Not available
Poor recruitment^a^	49 (45.4)	17 (32.7)	32 (57.1)	30 (61.2)	26 (53.1)	14 (28.6)	19 (38.8)
Organizational or strategic reasons	13 (12.0)	7 (13.5)	6 (10.7)	6 (46.2)	4 (30.8)	3 (23.1)	7 (53.9)
Futility	12 (11.2)	12 (23.1)	0	12 (100)	8 (66.7)	10 (83.3)	0
Harm	6 (5.6)	4 (7.7)	2 (3.6)	5 (83.3)	4 (66.7)	3 (50.0)	1 (16.7)
Benefit	5 (4.6)	3 (5.8)	2 (3.6)	4 (80.0)	2 (40.0)	3 (60.0)	1 (20.0)
External evidence	2 (1.9)	2 (3.8)	0	2 (100)	2 (100)	2 (100)	0
COVID-19	2 (1.9)	2 (3.8)	0	2 (100)	1 (50.0)	2 (100)	0
Lack of funding	2 (1.9)	1 (1.9)	1 (1.8)	1 (50.0)	1 (50.0)	1 (50.0)	1 (50.0)
Unclear	17 (15.7)	4 (7.7)	13 (23.2)	12 (70.6)	12 (70.6)	5 (29.4)	5 (29.4)

Abbreviation: RCTs, randomized clinical trials.

^a^ Twelve studies that stated slow recruitment as reason for discontinuation also mentioned another reason (ie, COVID-19 [n = 7], external evidence [n = 3], organizational or strategic reason [n = 2]).

In our multivariable analyses, industry-sponsored trials had lower odds of nonpublication (adjusted OR, 0.18 [95% CI, 0.10-0.33]) and discontinuation due to poor recruitment (adjusted OR, 0.32 [95% CI, 0.15-0.71]) (Table 4). Both associations were confirmed in our sensitivity analyses, including data from RCTs approved in 2012 (eTable 7 in Supplement 1) and accounting for potential cluster effects (eTable 8 in Supplement 1). RCT protocols with higher adherence to the SPIRIT reporting guidelines had lower odds of nonpublication of trial results (adjusted OR, 0.71 [95% CI, 0.57-0.89]). For the other assessed characteristics, no association was found.

**Table 4.** Factors Associated With Making Trial Results Available and Trial Discontinuation Due to Poor Recruitment

Characteristic	RCT availability/discontinuation status, No. (%)		Logistic regression			
			Univariable		Multivariable	
	Without results available (n=71) or discontinued due to poor recruitment (n=49)	With results available (n=276) or not discontinued due to poor recruitment (n=285)	OR (95% CI)	*P* value	AOR (95% CI)	*P* value
**Availability**						
Proportion of adequate SPIRIT reporting, median (IQR)^a^	0.68 (0.59-0.81)	0.76 (0.71-0.81)	0.65 (0.53-0.81)	<.001	0.71 (0.57-0.89)	.004
Planned target sample size, median (IQR)^b^	120 (54-260)	252 (120-509)	0.84 (0.75-0.95)	.005	0.92 (0.85-1.00)	.06
Placebo-controlled vs not placebo-controlled	28 (39.4)	124 (44.9)	0.80 (0.47-1.36)	.41	1.55 (0.82-2.90)	.18
Single-center vs multicenter	35 (49.3)	47 (17.0)	4.74 (2.70-8.30)	<.001	1.50 (0.75-2.99)	.25
Reported recruitment projection	19 (26.7)	62 (22.5)	1.26 (0.69-2.29)	.45	1.10 (0.56-2.13)	.79
Industry sponsorship	15 (21.1)	166 (60.1)	0.18 (0.10-0.33)	<.001	0.21 (0.10-0.44)	<.001
**Discontinuation**						
Proportion of adequate SPIRIT reporting, median (IQR)^a^	0.74 (0.68-0.83)	0.77 (0.70,0.81)^c^	1.01 (0.76-1.34)	.95	1.05 (0.79-1.40)	.76
Planned target sample size, median (IQR)^b^	216 (100-316)	232 (118-495)^c^	0.94 (0.87-1.02)	.14	0.96 (0.90-1.03)	.26
Placebo-controlled vs not placebo-controlled	25 (51.0)	122 (42.8)^c^	1.39 (0.76-2.55)	.29	1.96 (1.00-3.84)	.05
Single-center vs multicenter	16 (32.7)	58 (20.4)^c^	1.90 (0.98-3.68)	.06	1.02 (0.45-2.32)	.96
Reported recruitment projection	9 (18.4)	71 (24.9)^c^	0.68 (0.31-1.47)	.32	0.61 (0.28-1.36)	.27
Industry sponsorship	17 (34.7)	160 (56.1)^c^	0.42 (0.22-0.78)	.006	0.32 (0.15-0.71)	.005

Abbreviations: AOR, adjusted odds ratio; OR, odds ratio; RCT, randomized clinical trial; SPIRIT, Standard Protocol Items: Recommendations for Interventional Trials.

^a^ In increments of 10%.

^b^ In increments of 100.

^c^ Studies with unclear discontinuation status excluded (n = 13).

## Discussion

In 2005, the International Committee of Medical Journal Editors mandated prospective trial registration (ie, before enrolling the first participant) as a condition of publication.^18^ Our systematic review showed that, more than 10 years later, 1 of 4 non–industry-sponsored trials approved by a REC did not fulfill this requirement. In contrast, among industry-sponsored RCTs, only 7.8% of trials were not properly registered (ie, 2.8% not registered, 5.0% retrospectively registered). A study published in 2023 found that among Swiss trials receiving ethical approval between 2016 to 2020, 9% were unregistered.^19^ This number is slightly higher compared with our observed percentage of unregistered trials in Switzerland (6.4%) and does not indicate improvement over time (eTable 3 in Supplement 1). Hence, action should be taken to increase registration rates (eg, implementing controlling and enforcing regulations by ethical committees and publishing journals).

Our study findings indicated that trial registries are an important approach to make trial results available. Among the RCTs that did not publish results in a peer-reviewed journal, 27.6% reported results in a trial registry (63.4% industry-sponsored RCTs, 1.8% non–industry-sponsored RCTs). These findings suggest that it is crucial to include trial registries when extracting outcome data in systematic reviews and meta-analyses.^20,21^ However, as in a previous study of RCTs approved in 2012 by members of our team,^9^ investigators from non–industry-sponsored trials approved in 2016 rarely published results in trial registries (ie, only 17 of 166 [10.2%] in 2016). In Switzerland, it has been mandatory since March 2025 to publish trial results in a registry within 1 year of study completion.^22^ Similar laws exist already in the European Union for trials testing medical products (ie, drugs, vaccines, or biological products).^23^ Further evaluations will be required to assess whether these requirements can be enforced or whether more pragmatic solutions can be implemented. One strategy to reduce administrative burden among trialists would be to allow registries to link to a journal publication of trial results. However, this would require that all end points (including adverse events) are completely reported in published articles.^21,24,25,26^ Availability of trials results has shown substantial improvement during the past 2 decades, with 87% of trials approved in 2012 providing results at a 10-year follow-up and comparable results for RCTs approved in 2016. The most substantial progress occurred during the period when trial registration became mandatory.^18^ Moving forward, it will be important to explore how to ensure that all trial results are made publicly available.

Another major persistent challenge is that approximately one-third of all RCTs are prematurely discontinued, mainly due to poor recruitment of participants. Our repeated investigation of trial protocols approved from 2000 to 2016 showed no improvement over time and further showed that trial discontinuation was associated with nonpublication of trial results. One strategy to address this lack of improvement would be for funders to require completion of (internal) pilot trials demonstrating feasibility as a precondition of funding definitive trials.^27^ Emerging trial designs, such as adaptive platform trials or Trials within Cohorts, may also help by promoting more efficient participant recruitment.^28,29,30^ Another approach could be to design pragmatic RCTs that follow clinical routine as much as possible.^31^ However, these RCTs using a pragmatic approach might be endangered by overly restrictive regulatory requirements.^32,33,34^

Similar to our observations, a recently published Cochrane methodology review, which included 204 research reports, found that results of multicenter RCTs were more often made publicly available than those of single-center RCTs.^35^ However, in contradiction to our findings, the authors also reported that industry-sponsored trials were less often published compared with non–industry-sponsored trials.^35^ This result may have been affected by the inclusion of several older studies; the present analysis found that industry-sponsored RCTs greatly improved during the last decade in making results publicly available (eTable 4 in Supplement 1).

### Strengths and Limitations

This study has several strengths. Having access to study protocols approved by RECs in 4 different countries increased the generalizability of our findings. Collaborating with the same ethics committees as in previous studies conducted by members of our team and using the same methods^8,9^ enabled us to make comparisons over time. Merging our data with those from 2012 increased our power to assess factors associated with nonpublication and trial discontinuation due to poor recruitment.

This study has several limitations. First, it is plausible that some of the 20 RCTs (5.8%) for which we could not find a registration number did not begin recruitment of participants, leading to overestimation of the percentage of unregistered RCTs. Second, since our sample consisted of RCTs approved in 4 countries in 2016, the rates of nonregistration, discontinuation, and nonpublication may be different in other countries or among trial protocols approved more recently.

## Conclusions

Inadequate trial registration (ie, nonregistration and retrospective registration), premature discontinuation due to poor recruitment, and nonpublication of RCT results remain major challenges in clinical research. The results of our systematic review indicated that industry-sponsored trials performed better in these aspects, whereas non–industry-sponsored trials showed a greater need for improvement. We recommend that granting agencies mandate completion of a successful (internal) pilot trial before funding full definitive trials and require investigators to publish their findings publicly as a condition of funding. When it comes to sharing trial results in registries, it is important to assess the feasibility for non–industry-sponsored trials, particularly given the often high administrative burden on investigators. More pragmatic solutions—such as linking registry entries directly to journal publications—may offer a viable alternative that should be considered by responsible stakeholders.

## Notes

Supplement 1. **eMethods.** Sensitivity analyses **eTable 1.** Baseline characteristics of included randomized clinical trials, stratified by country of ethical approval **eTable 2.** Baseline characteristics of included randomized clinical trials in the current study and in the meta research studies assessing protocols receiving ethical approval in 2000-2003 [4], and 2012 [1] **eTable 3.** Non-registration, discontinuation, and non-publication of randomized clinical trials receiving ethical approval in 2016, stratified by country of ethical approval **eTable 4.** Non-publication and discontinuation in protocols approved by ethical committees in 2016 compared to protocols approved between 2000 to 2003 [4] and in 2012 [1] **eTable 5.** Non-publication of randomized trials approved by ethical committees in 2016 compared to protocols approved in 2012, using an adjusted follow-up time for trials from 2012 **eTable 6.** Association between discontinuation of randomized clinical trials and non-publishing of study results **eTable 7.** Sensitivity analysis to assess factors associated with making trial results available and trial discontinuation due to poor recruitment using multivariable logistic regression, including RCTs receiving ethical approval in 2012 or 2016 (N = 673) **eTable 8.** Sensitivity analysis to assess factors associated with making trial results available and trial discontinuation due to poor recruitment using conservative cluster-robust standard errors with covariance matrix estimation HC3 bias adjustment [2, 3] to account for potential country-level correlation in the data

Supplement 2. **Nonauthor Collaborators.** The ASPIRE Study Group

Supplement 3.
Data Sharing Statement
