# Supplementary material for: Nonregistration, Discontinuation, and Nonpublication of Randomized Trials: A Systematic Review
Source: JAMA Netw Open. 2025 Sep 3;8(9):e2524440. doi: 10.1001/jamanetworkopen.2025.24440 (PMC12409572; doi:10.1001/jamanetworkopen.2025.24440)
Supplement: Supplement 1. — eMethods. Sensitivity analyses eTable 1. Baseline characteristics of included randomized clinical trials, stratified by country of ethical approval eTable 2. Baseline characteristics of included randomized clinical trials in the current study and in the meta research studies assessing protocols receiving ethical approval in 2000-2003 [4], and 2012 [1] eTable 3. Non-registration, discontinuation, and non-publication of randomized clinical trials receiving ethical approval in 2016, stratified by country of ethical approval eTable 4. Non-publication and discontinuation in protocols approved by ethical committees in 2016 compared to protocols approved between 2000 to 2003 [4] and in 2012 [1] eTable 5. Non-publication of randomized trials approved by ethical committees in 2016 compared to protocols approved in 2012, using an adjusted follow-up time for trials from 2012 eTable 6. Association between discontinuation of randomized clinical trials and non-publishing of study results eTable 7. Sensitivity analysis to assess factors associated with making trial results available and trial discontinuation due to poor recruitment using multivariable logistic regression, including RCTs receiving ethical approval in 2012 or 2016 (N = 673) eTable 8. Sensitivity analysis to assess factors associated with making trial results available and trial discontinuation due to poor recruitment using conservative cluster-robust standard errors with covariance matrix estimation HC3 bias adjustment [2, 3] to account for potential country-level correlation in the data [file jamanetwopen-e2524440-s001.pdf]

Supplementary Online Content

Speich B, Taji Heravi A, Schönenberger C, et al. Nonregistration, discontinuation, and nonpublication of randomized trials: a systematic review. *JAMA Netw Open*. 2025;8(9):e2524440. doi:10.1001/jamanetworkopen.2025.24440

eMethods. Sensitivity analyses

eTable 1. Baseline characteristics of included randomized clinical trials, stratified by country of ethical approval

eTable 2. Baseline characteristics of included randomized clinical trials in the current study and in the previous studies assessing protocols receiving ethical approval in 2000 to 2003 [4], and 2012 [1]

eTable 3. Non-registration, discontinuation, and non-publication of randomized clinical trials receiving ethical approval in 2016, stratified by country of ethical approval

eTable 4. Non-publication and discontinuation in protocols approved by ethical committees in 2016 compared to protocols approved between 2000 to 2003 [4] and in 2012 [1]

eTable 5. Non-publication of randomized trials approved by ethical committees in 2016 compared to protocols approved in 2012, using an adjusted follow-up time for trials from 2012

eTable 6. Association between discontinuation of randomized clinical trials and non-publishing of study results

eTable 7. Sensitivity analysis to assess factors associated with making trial results available and trial discontinuation due to poor recruitment using multivariable logistic regression, including RCTs receiving ethical approval in 2012 or 2016 (N=673)

eTable 8. Sensitivity analysis to assess factors associated with making trial results available and trial discontinuation due to poor recruitment using conservative cluster-robust standard errors with covariance matrix estimation HC3 bias adjustment [2, 3] to account for potential country-level correlation in the data

eReferences.

This supplementary material has been provided by the authors to give readers additional information about their work.

© 2025 Speich B et al. *JAMA Network Open*.

eMethods. Sensitivity analyses

i) To increase the power and explore association over time, we repeated our regression analyses including also data from our study on trial protocols approved in 2012 (year of approval was added as a variable in the regression model) [1].

ii) In a sensitivity analysis, we obtained cluster-robust standard errors using covariance matrix estimation with HC3 bias adjustment to account for potential country-level correlation in the data [2, 3].

© 2025 Speich B et al. *JAMA Network Open*.

eTable 1. Baseline characteristics of included randomized clinical trials, stratified by country of ethical approval

	Switzerland (n=188)[a]	United Kingdom (n=97)[a]	Germany (n=33)[a]	Canada (n=29)[a]	All RCTs (n=347)
**Planned sample size, median (IQR)**	199 (87-498)	232 (96-390)	308 (180-628)	270 (154-400)	220 (102-450)
**Proportion of adequately reported SPIRIT items in protocol, median (IQR)**	0.79 (0.72-0.83)	0.73 (0.67-0.78)	0.72 (0.64-0.77)	0.72 (0.68-0.79)	0.76 (0.68-0.81)
**Single centre vs. multicentre**					
**Single centre**	56 (29.8%)	20 (20.6%)	3 (9.1%)	3 (10.3%)	82 (23.6%)
**Multicentre**	132 (70.2%)	77 (79.4%)	30 (90.9%)	26 (89.7%)	265 (76.4%)
**Study design**					
**Parallel**	169 (89.9%)	93 (95.9%)	33 (100.0%)	27 (93.1%)	322 (92.8%)
**Crossover**	10 (5.3%)	2 (2.1%)	0 (0.0%)	1 (3.5%)	13 (3.8%)
**Factorial**	4 (2.1%)	1 (1.0%)	0 (0.0%)	0 (0.0%)	5 (1.4%)
**Cluster**	4 (2.1%)	0 (0.0%)	0 (0.0%)	0 (0.0%)	4 (1.2%)
**Split body**	1 (0.5%)	1 (1.0%)	0 (0.0%)	1 (3.5%)	3 (0.9%)
**Placebo controlled**	77 (41.0%)	43 (44.3%)	18 (54.5%)	14 (48.3%)	152 (43.8%)
**Recruitment-rate reported in protocol**	29 (15.4%)	38 (39.2%)	6 (18.2%)	8 (27.6%)	81 (23.3%)
**Type of sponsor**					
**Industry**	79 (42.0%)	60 (61.9%)	26 (78.8%)	16 (55.2%)	181 (52.2%)
**Non-industry**	109 (58.0%)	37 (38.1%)	7 (21.2%)	13 (44.8%)	166 (47.8%)

Abbreviations: RCT=randomized clinical trial; IQR=interquartile range

[a]The following ethical committees were involved: Switzerland: All 7 national ethical committees; United Kingdom: Bristol office of the UK National Research Ethics Service which is overseeing 19 RECs across the United Kingdom; Germany: ethical committee of Freiburg; Canada: ethical committee of Hamilton

© 2025 Speich B et al. *JAMA Network Open*.

eTable 2. Baseline characteristics of included randomized clinical trials in the current study and in the meta research studies assessing protocols receiving ethical approval in 2000-2003 [4], and 2012 [1]

	RCTs approved 2000-2003 [4] (n=894)	RCTs approved in 2012 [1] (n=326)	RCTs in approved in 2016 (n=347)
**Planned sample size, median (IQR)**	260 (100-610)[a]	250 (100-600)	220 (102-450)
**Proportion of adequately reported SPIRIT items in protocol, median (IQR)[b]**	-	0.69 (0.61-0.77)	0.76 (0.68-0.81)
**Single centre vs. multicentre[c]**			
**Single centre**	149 (16.6%)	60 (18.4%)	82 (23.6%)
**Multicentre**	741 (82.9%)	266 (81.6%)	265 (76.4%)
**Study design**			
**Parallel**	822 (92.0%)	296 (90.8%)	322 (92.8%)
**Crossover**	41 (4.6%)	13 (4.0%)	13 (3.8%)
**Factorial**	14 (1.6%)	10 (3.1%)	5 (1.4%)
**Cluster**	12 (1.3%)	4 (1.2%)	4 (1.2%)
**Split body**	3 (0.3%)	0 (0.0%)	3 (0.9%)
**Other/Unclear**	2 (0.2%)	3 (0.9%)	152 (43.8%)
**Placebo controlled**	346 (38.7%)	131 (40.2%)	81 (23.3%)
**Type of sponsor**			
**Industry**	551 (61.6%)	179 (54.9%)	181 (52.2%)
**Non-industry**	343 (38.4%)	147 (45.1%)	166 (47.8%)
**Country of ethical approval**			
**Switzerland**	444 (49.7%)	165 (50.6%)	188 (54.2%)
**United Kingdom**	0 (0.0%)	89 (27.3%)	97 (28.0%)
**Germany**	272 (30.4%)	37 (11.4%)	33 (9.5%)
**Canada**	178 (19.9%	35 (10.7%)	29 (8.4%)

[a]12 trial protocols with missing target sample size excluded.
[b] Adherence to SPIRIT reporting guidelines was not assessed for study protocols approved in 2000-2003.
[c] For 4 protocols approved in 2000-2003 it remained unclear how many study centres were involved.
Abbreviations: RCT=randomized clinical trial; IQR=interquartile range

© 2025 Speich B et al. *JAMA Network Open*.

eTable 3. Non-registration, discontinuation, and non-publication of randomized clinical trials receiving ethical approval in 2016, stratified by country of ethical approval

	Switzerland (n=188)[a]	United Kingdom (n=97)[a]	Germany (n=33)[a]	Canada (n=29)[a]	All RCTs (n=347)
**Registration status**					
Registered	176 (93.7%)	93 (95.9%)	31 (93.9%)	27 (93.1%)	327 (94.2%, 91.2-96.4%)
Prospectively registered	155 (82.4%)	84 (86.6%)	30 (90.9%)	25 (86.2%)	294 (84.7%, 80.5-88.3%)
Retrospectively registered	21 (11.2%)	9 (9.3%)	1 (3.0%)	2 (6.9%)	33 (9.5%, 6.7-13.1%)
Not registered	12 (6.4%)	4 (4.1%)	2 (6.1%)	2 (6.9%)	20 (5.8%, 3.6-8.8%)
**Completion status**					
Completed	117 (62.4%)	69 (71.1%)	19 (57.6%)	21 (72.4%)	226 (65.1%, 59.9-70.1%)
Discontinued	65 (34.6%)	25 (25.8%)	12 (36.4%)	6 (20.7%)	108 (31.1%, 26.3-36.3%)
Unclear	6 (3.2%)	3 (3.1%)	2 (6.1%)	2 (6.9%)	13 (3.8%, 2.0-6.3%)
**Results availability**					
At any source (peer-reviewed publication or on trial registry)	144 (76.6%)	79 (81.4%)	31 (93.9%)	22 (75.9%)	276 (79.5%, 74.9-83.7%)
Peer reviewed publication	135 (71.8%)	70 (72.2%)	26 (78.8%)	18 (62.1%)	249 (71.8, 66.7-76.4%)
In trial registry	68 (36.2%)	59 (60.8%)	25 (75.8%)	18 (62.1%)	170 (49.0% 43.6-54.4%)
Results not available (neither as publication nor in trial registry	44 (23.4%)	18 (18.6%)	2 (6.1%)	7 (24.1%)	71 (20.5%, 16.3-25.1%)
**Neither registered nor published**	10 (5.3%)	4 (4.1%)	2 (6.1%)	2 (6.9%)	18 (5.2%, 3.1-8.1%)
**Not published in journal but registered[a]**	43 (81.1%)	23 (85.2%)	5 (71.4%)	9 (81.8%)	80 (81.6%, 72.5-88.7%)
**Not published in journal but results available in registry[b]**	9 (17.0%)	9 (33.3%)	5 (71.4%)	4 (36.4%)	27 (27.6%, 19.0-37.5%)

[a]The following ethical committees were involved: Switzerland: All 7 national ethical committees; United Kingdom: Bristol office of the UK National Research Ethics Service which is overseeing 19 RECs across the United Kingdom; Germany: ethical committee of Freiburg; Canada: ethical committee of Hamilton
[b]Only a subsample of 98 trials considered (53 Switzerland, 27 United Kingdom, 7 Germany, 11 Canada) which were not published in a peer reviewed journal

© 2025 Speich B et al. *JAMA Network Open*.

eTable 4. Non-publication and discontinuation in protocols approved by ethical committees in 2016 compared to protocols approved between 2000 to 2003 [4] and in 2012 [1]

	Study-protocols approved 2000-2003 [4]			Study-protocols approved in 2012 [1]			Study-protocols approved in 2016		
	Industry sponsored RCTs	Non-industry sponsored RCTs	All RCTs	Industry sponsored RCTs	Non-industry sponsored RCTs	All RCTs	Industry sponsored RCTs	Non-industry sponsored RCTs	All RCTs
**Registration status**[a]									
Registered	-	-	-	175/179 (97.8%)	132/147 (89.8%)	307/326 (94.2%)	176/181 (97.2%)	151/166 (91.0%)	327/347 (94.2%)
Prospectively registered	-	-	-	164/179 (91.6%)	110/147 (74.8%)	274/326 (84.0%)	167/181 (92.3)	127/166 (76.5%)	294/347 (84.7%
Retrospectively registered	-	-	-	10/179 (5.6%)	22/147 (15.0%)	33/326 (10.1%)	9/181 (5.0%	24/166 (14.5%)	33/347 (9.5%)
Not registered	-	-	-	4/179 (2.2%)	15/147 (10.2%)	19/326 (5.9%)	5/181 (2.8%	15/166 (9.0%)	20/347 (5.8%)
**Completion status**									
Completed	394/551 (71.5%)	181/343 (52.8%)	575/894 (64.3%)	119/179 (66.5%)	84/147 (57.1%)	203/326 (62.3%)	125/181 (69.1%)	101/166 (60.8%)	226/347 (65.1%)
Discontinued	119/551 (21.6%)	130/343 (37.9%)	249/894 (27.9%)	57/179 (31.8%)	41/147 (27.9%)	98/326 (30.1%)	52/181 (28.7%)	56/166 (33.7%)	108/347 (31.1%)
Unclear	38/551 (6.9%)	32/343 (9.3%)	70/894 (7.8%)	3/179 (1.7%)	22/147 (15.0%)	25/326 (7.7%)	4/181 (2.2%=	9/166 (5.4%)	13/347 (3.8%)
**Results availability**									
At any source (peer-reviewed publication or on trial registry)[a]	336/551 (61.0%)	194/343 (56.6%)	530/894 (59.3%)	172/179 (96.1%)	112/147 (76.2%)	284/326 (87.1%)	166/181 (91.7%)	110/166 (66.3%)	276/347 (79.5%)
Peer reviewed publication	336/551 (61.0%)	194/343 (56.6%)	530/894 (59.3%)	146/179 (81.6%)	100/147 (74.8%)	256/326 (78.5%)	140/181 (77.4%)	109/166 (65.7%)	249/347 (71.8)
In trial registry[a]	-	-	-	150/179 (83.8%)	23/147 (15.7%)	173/326 (53.1%)	153/181 (84.5%)	17/166 (10.2%)	170/347 (49.0%)
**Reasons for discontinuation**									
Poor recruitment[b]	40/119 (34%)	60/130 (46%)	100/249 (40%)	16/57 (28%)	20/41 (49%)	36/98 (37%)	17/52 (32.7%)	32/56 (57.1%)	49/108 (45.4%)
Futility	25/119 (21%)	12/130 (9%)	37/249 (15%)	15/57 (26%)	1/41 (2%)	16/98 (16%)	12/52 (23.1%)	6/56 (10.7%)	13/108 (12.0%)
Organisational/strategic reasons	20/119 (17%)	16/130 (12%)	36/249 (14%)	6/57 (11%)	0/41 (0%)	6/98 (6%)	7/52 (13.5%)	0/56 (0.0%)	12/108 (11.2%)
Harm	17/119 (14%)	7/130 (5%)	24/249 (10%)	5/57 (9%)	1/41 (2%)	6/98 (6%)	4/52 (7.7%)	2/56 (3.6%)	6/108 (5.6%)
Benefit	2/119 (2%)	7/130 (5%)	9/249 (4%)	2/57 (4%)	1/41 (2%)	3/98 (3%)	3/52 (5.8%)	2/56 (3.6%)	5/108 (4.7%)
External evidence	6/119 (5%)	2/130 (2%)	8/249 (3%)	0/57 (0%)	3/41 (7%)	3/98 (3%)	2/52 (3.9%)	0/56 (0.0%)	2/108 (1.9%)
Limited resources	1/119 (1%)	4/130 (3%)	5/249 (2%)	0/57 (0%)	1/41 (2%)	1/98 (1%)	1/52 (1.9%)	1/56 (1.8%)	2/108 (1.9%)
Unclear	6/119 (5%)	18/130 (14%)	24/249 (10%)	13/57 (23%)	14/41 (34%)	27/98 (28%)	4/52 (7.7%)	13/56 (23.2%)	17/108 (15.7%)
Other	2/119 (2%)	4/130 (3%)	6/249 (2%)	0/57 (0%)	0/41 (0%)	0/98 (0%)	2/52 (3.9%)	0/55 (0.0%)	2/108 (1.9%)

[a]Trial registration were not established in 2000-2003; hence registration was not assessed for RCT protocols approved in 2000-20003 and peer reviewed publication was the only source considered for sharing results.

Abbreviations: RCT=randomized clinical trial

© 2025 Speich B et al. *JAMA Network Open*.

eTable 5. Non-publication of randomized trials approved by ethical committees in 2016 compared to protocols approved in 2012, using an adjusted follow-up time for trials from 2012

	Study-protocols approved in 2012		
	Industry sponsored RCTs	Non-industry sponsored RCTs	All RCTs
**Results availability for all RCTs approved in 2016 with a follow-up time of ~8.5 years**	166/181 (91.7%)	110/166 (66.3%)	276/347 (79.5%)
**Results availability for all RCTs approved in 2012 adjusting the follow-up time for RCTs from 2012 to ~8.5 years***	168/179 (93.9%)	99/147 (67.4%)	267/326 (81.9%)
**Results availability for all RCTs approved in 2012 adjusting the follow-up time for RCTs from 2012 to ~10 years**	172/179 (96.1%)	112/147 (76.2%)	284/326 (87.1%)

*excluding for 2012 results which were made publicly available after the 1.8.2020, in order to provide the same follow-up duration as for trials approved in 2016.
Abbreviations: RCT=randomized clinical trial

eTable 6. Association between discontinuation of randomized clinical trials and non-publishing of study results

	Completed RCTs (n=226)[a]	Discontinued RCTs (n=108)[a]	Odds ratio (95% Confidence Interval)	*p-value*
Results available at any source (peer-reviewed publication or trial registry)	202 (89.4%)	74 (68.5%)	4.87 (2.07-7.27)	<0.001
Results available as a peer reviewed publication	189 (83.6%)	60 (55.6%)	4.09 (2.36-7.09)	<0.001
Results available in trial register	127 (56.2%)	43 (39.8%)	1.94 (1.19-3.18)	0.005

[a] Randomized clinical trials with unclear discontinuation status were excluded (n=13)
Abbreviations: RCT=randomized clinical trial

© 2025 Speich B et al. *JAMA Network Open*.

eTable 7. Sensitivity analysis to assess factors associated with making trial results available and trial discontinuation due to poor recruitment using multivariable logistic regression, including RCTs receiving ethical approval in 2012 or 2016 (N=673)

**Characteristics**			**Multivariable**		
			**OR**	**95% CI**	***P-value***
**Non-availability of trial results (considering peer-reviewed publication and trial registries)**[a]	**RCT results not available (n=130)**	**RCT results available (peer reviewed journal or trial registry) (n=543)**			
Proportion of adequate SPIRIT reporting, median (IQR)[b]	0.67 (0.54-0.79)	0.74 (0.66-0.80)	0.74	0.63-0.87	<0.001
Planned target sample size, median (IQR)[c]	120 (54-222)	286 (120-600)	1.00	0.98-1.01	0.57
Placebo controlled (vs not placebo controlled)	47/130 (36.2%)	236/543 (43.5%)	1.44	0.91-2.28	0.18
Single-centre (vs multicentre)	57/130 (43.9%)	85/543 (15.7%)	1.64	1.00-2.69	0.05
Reported recruitment projection	40/130 (30.8%)	140/543 (25.8%)	1.26	0.79-2.01	0.34
Industry sponsorship	26/130 (20.0%)	334/543 (61.5%)	0.21	0.13-0.37	<0.001
Approval in 2016 (vs 2012)	71/130 (54.6%)	276/543 (50.8%)	1.41	0.90-2.21	0.13
**Discontinued due to poor recruitment**[d]	**RCTs discontinued due to poor recruitment (n=85)**[d]	**RCTs not discontinued due to poor recruitment (n=550)**[d]			
Proportion of adequate SPIRIT reporting, median (IQR)[b]	0.72 (0.65-0.81)	0.73 (0.66-0.80))	1.05	0.84-1.30	0.67
Planned target sample size, median (IQR)[c]	200 (90-334)	269 (110-600)	0.96	0.91-1.00	0.07
Placebo controlled (vs not placebo controlled)	40/85 (47.1%)	231/550 (42.0%)	1.61	0.98-2.65	0.06
Single-centre (vs multicentre)	24/85 (28.2%)	97/550 (17.6%)	1.00	0.53-1.86	0.99
Reported recruitment projection	20/85 (23.5%)	149/550 (27.1%)	0.78	0.45-1.38	0.40
Industry sponsorship	33/85 (38.8%)	320/550 (58.2%)	0.40	0.23-0.71	0.002
Approval in 2016 (vs 2012)	49/85 (57.7%)	285/550 (51.8%)	1.07	0.65-1.77	0.80

[a] Excluding for 2012 results which were made publicly available after the 1.8.2020, in order to provide the same follow-up duration as for trials approved in 2016.
[b] In increments of 10%
[c] In increments of 100
[d] Studies with unclear discontinuation status excluded (n=38)
Abbreviations: OR=odds ratio; CI= confidence Interval; IQR=interquartile range; RCT=randomized clinical trial

© 2025 Speich B et al. *JAMA Network Open.*

eTable 8. Sensitivity analysis to assess factors associated with making trial results available and trial discontinuation due to poor recruitment using conservative cluster-robust standard errors with covariance matrix estimation HC3 bias adjustment [2, 3] to account for potential country-level correlation in the data

[a] In increments of 10%

**Characteristics**			**Multivariable**		
			**OR**	**95% CI**	***P-value***
**Non-availability of trial results (considering peer-reviewed publication and trial registries)**	**RCT results not available (n=71)**	**RCT results available (peer-reviewed journal or trial registry) (n=276)**			
Proportion of adequate SPIRIT reporting, median (IQR)[a]	0.68 (0.59, 0.81)	0.76 (0.71, 0.81)	0.71	0.38-1.32	0.28
Planned target sample size, median (IQR)[b]	120 (54, 260)	252 (120, 509)	0.92	0.51-1.69	0.80
Placebo controlled vs not placebo controlled (%)	28/71 (39.4%)	124/276 (44.9%)	1.55	0.14-16.83	0.72
Single-center vs multicenter (%)	35/71 (49.3%)	47/276 (17.0%)	1.50	0.80-2.79	0.21
Reported recruitment projection (%)	19/71 (26.7%)	62/276 (22.5%)	1.10	0.43-2.82	0.85
Industry sponsorship (%)	15/71 (21.1%)	166/276 (60.1%)	0.21	0.04-0.98	0.05
**Discontinued due to poor recruitment**	**RCTs discontinued due to poor recruitment (n=49)**	**RCTs not discontinued due to poor recruitment (n=285)[c]**			
Proportion of adequate SPIRIT reporting, median (IQR)a	0.74 (0.68, 0.83)	0.77 (0.70,0.81)	1.05	0.70-1.57	0.83
Planned target sample size, median (IQR)b	216 (100, 316)	232 (118, 495)	0.96	0.92-1.01	0.19
Placebo controlled vs not placebo controlled (%)	25/49 (51.0%)	122/285 (42.8%)	1.96	0.22-17.10	0.54
Single-center vs multicenter (%)	16/49 (32.7%)	58/285 (20.4%)	1.02	0.61-1.70	0.94
Reported recruitment projection (%)	9/49 (18.4%)	71/285 (24.9%)	0.61	0.34-1.09	0.10
Industry sponsorship (%)	17/49 (34.7%)	160/285 (56.1%)	0.32	0.16-0.64	0.001

[b] In increments of 100
[c] Studies with unclear discontinuation status excluded (n=13)
Abbreviations: OR=odds ratio; CI= confidence Interval; IQR=interquartile range; RCT=randomized clinical trial

© 2025 Speich B et al. *JAMA Network Open.*

eReferences.

1. Speich B, Gryaznov D, Busse JW, Gloy VL, Lohner S, Klatte K, et al. Nonregistration, discontinuation, and nonpublication of randomized trials: A repeated metaresearch analysis. PLoS Med. 2022;19(4):e1003980.
2. Zeileis A. Econometric Computing with HC and HAC Covariance Matrix Estimators. Journal of Statistical Software. 2004;11(10):1 - 17. doi: 10.18637/jss.v011.i10.
3. Zeileis A and Thomas L. 2004. Sandwich: Robust Covariance Matrix Estimators. The R Foundation. https://doi.org/10.32614/cran.package.sandwich [accessed: 20. February 2025].
4. Kasenda B, von Elm E, You J, Blumle A, Tomonaga Y, Saccilotto R, et al. Prevalence, characteristics, and publication of discontinued randomized trials. JAMA. 2014;311(10):1045-51.

© 2025 Speich B et al. *JAMA Network Open*.
