# Supplementary material for: Nonregistration, Discontinuation, and Nonpublication of Randomized Trials: A Systematic Review
Source: JAMA Netw Open. 2025 Sep 3;8(9):e2524440. doi: 10.1001/jamanetworkopen.2025.24440 (PMC12409572; doi:10.1001/jamanetworkopen.2025.24440)
Supplement: Supplement 2. — Nonauthor Collaborators. The ASPIRE Study Group [file jamanetwopen-e2524440-s002.pdf]

Supplemental Online Content: Nonauthor Collaborators
*First name, last name, and suffix (if applicable) are required and will appear in PubMed.

*Group Name: The Adherence to SPIrit REcommendations (ASPIRE) group							
***First Name and Middle Initial(s)**	***Last Name**	***Suffix (eg, Jr, III)**	Academic Degrees	Institution	Location (city, state/province, country)	Role or Contribution, eg, chair, principal investigator	Group (if more than 1 Group listed in the byline) and/or Subgroup (eg, Steering Committee)
Benjamin	Speich		PhD	University Hospital Basel	Basel, Switzerland	Lead of Sub-projects; Study design, data collection	
Dmitry	Gryaznov		MD, MSC	University Hospital Basel	Basel, Switzerland	Former PhD student; Study design, project managment, data	
Belinda	von Niederhäusern		PhD	Roche	Grenzach-Wyhlen, Germany	Former PhD student; Study design, project managment, data collection	
Benjamin	Kasenda		PhD	University Hospital Basel	Basel, Switzerland	Collaborator; Project design of ASPIRE and other sub-	
Elena	Ojeda-Ruiz		MD, MSC	Madrid Salud	Madrid, Spain	Collaborator; Data collection for ASPIRE	
Anette	Blümle		PhD	University of Freiburg	Freiburg, Germany	Lead of sub-projects; Study design, data collection	
Stefan	Schandelmaier		MD, PhD	University Hospital Basel	Basel, Switzerland	Collaborator; Project design of ASPIRE and other sub-	
Dominik	Mertz		Prof	McMaster University	Hamilton, Canada	Collaborator; Study design, data collection	
Ayodele	Odutayo		MD, PhD	University of Toronto	Ontario, Canada	Collaborator; Study design, data collection	
Yuki	Tomonaga		PhD	University of Zürich	Zürich, Switzerland	Collaborator; Data collection for ASPIRE	
Alain	Amstutz		MD, PhD	University Hospital Basel	Basel, Switzerland	Collaborator; Project design of ASPIRE and other sub-projects	
Christiane	Pauli-Magnus		Prof	University Hospital Basel	Basel, Switzerland	Collaborator; Project design of ASPIRE and other sub-projects	
Viktoria	Gloy		PhD	Federal Office of Public Health	Bern, Switzerland	Collaborator; Project design of ASPIRE and other sub-projects	

*First name, last name, and suffix (if applicable) are required and will appear in PubMed.

***First Name and Middle Initial(s)**	***Last Name**	***Suffix (eg, Jr, III)**	Academic Degrees	Institution	Location (city, state/province, country)	Role or Contribution, eg, chair, principal investigator	Group (if more than 1 Group listed in the byline) and/or Subgroup (eg, Steering Committee)
Szimonetta	Lohner		MD, PhD	University of Pécs	Pécs, Hungary	Lead of sub-projects; Study design, data collection	
Karin	Bischoff		MSc	University of Freiburg	Freiburg, Germany	Collaborator; Project design of ASPIRE and other sub-projects	
Katharina	Wollmann		MSc	University of Freiburg	Freiburg, Germany	Collaborator; Project design of ASPIRE and other sub-projects	
Laura	Rehner		MSc	University Greifswald	Greifswald, Germany	Collaborator; Project design of ASPIRE and other sub-projects	
Joerg J	Meerpohl		Prof	University of Freiburg	Freiburg, Germany	Collaborator; Project design of ASPIRE and other sub-projects	
Alain	Nordmann		Prof	University Hospital Basel	Basel, Switzerland	Collaborator; Project design of ASPIRE and other sub-projects	
Katharina	Klatte		PhD	University Hospital Basel	Basel, Switzerland	Collaborator; Project design of ASPIRE and other sub-projects	
Nilabh	Ghosh		PhD	Hamilton Company	Hamilton, Canada	Collaborator; Project design of ASPIRE and other sub-projects	
Ala	Taji Heravi		PhD	University Hospital Basel	Basel, Switzerland	Lead of Sub-projects; Study design, data collection	
Jacqueline	Wong		PhD	McMaster University	Hamilton, Canada	Collaborator; Project design of ASPIRE and other sub-projects	
Ngai	Chow		PhD	McMaster University	Hamilton, Canada	Collaborator; Project design of ASPIRE and other sub-projects	

Supplemental Online Content: Nonauthor Collaborators
*First name, last name, and suffix (if applicable) are required and will appear in PubMed.

***First Name and Middle Initial(s)**	***Last Name**	***Suffix (eg, Jr, III)**	Academic Degrees	Institution	Location (city, state/province, country)	Role or Contribution, eg, chair, principal investigator	Group (if more than 1 Group listed in the byline) and/or Subgroup (eg, Steering Committee)
Patrick	Jiho Hong		PhD	McMaster University	Hamilton, Canada	Collaborator; Project design of ASPIRE and other sub-projects	
Kimberly	Mc Cord		PhD	Boston Children's Hospital and Harvard Medical School	Boston MA; USA	Collaborator; Project design of ASPIRE and other sub-projects	
Sirintip	Sricharoenchai		PhD	University Hospital Basel	Basel, Switzerland	Collaborator; Project design of ASPIRE and other sub-projects	
Jason W	Busse		Prof	McMaster University	Hamilton, Canada	Collaborator; Project design of ASPIRE and other sub-projects	
Lukas	Kübler		MD	University Hospital Basel	Basel, Switzerland	Collaborator; Project design of ASPIRE and other sub-projects	
Pooja	Gandhi		PhD	University of Alberta	Edmonton, AB, Canada	Collaborator; Project design of ASPIRE and other sub-projects	
Zsuzsanna	Kontar		PhD	University of Pécs	Pécs, Hungary	Collaborator; Project design of ASPIRE and other sub-projects	
Julia	Hüllstrung		BSc	University Hospital Basel	Basel, Switzerland	Collaborator; Project design of ASPIRE and other sub-projects	
Mona	Elafy		BSc	University Hospital Basel	Basel, Switzerland	Collaborator; Project design of ASPIRE and other sub-projects	
Arnav	Agarwal		MD	McMaster University	Hamilton, Canada	Collaborator; Project design of ASPIRE and other sub-projects	
Ramon	Saccilotto		MD	University Hospital Basel	Basel, Switzerland	Collaborator; Project design of ASPIRE and other sub-projects	

Supplemental Online Content: Nonauthor Collaborators
*First name, last name, and suffix (if applicable) are required and will appear in PubMed.

***First Name and Middle Initial(s)**	***Last Name**	***Suffix (eg, Jr, III)**	Academic Degrees	Institution	Location (city, state/province, country)	Role or Contribution, eg, chair, principal investigator	Group (if more than 1 Group listed in the byline) and/or Subgroup (eg, Steering Committee)
Alexandra	Griessbach		PhD	University Hospital Basel	Basel, Switzerland	Collaborator; Project design of ASPIRE and other sub-projects	
Christof	Schönenberger		MD	University Hospital Basel	Basel, Switzerland	Collaborator; Project design of ASPIRE and other sub-projects	
Matthias	Schwenkglenks		Prof	University of Basel	Basel, Switzerland	Collaborator; Project design of ASPIRE and other sub-projects	
Giusi	Moffa		Prof	University of Basel	Basel, Switzerland	Collaborator; Project design of ASPIRE and other sub-projects	
Lars G	Hemkens		Prof	University Hospital Basel	Basel, Switzerland	Collaborator; Project design of ASPIRE and other sub-projects	
Sally	Hopewell		Prof	University of Oxford	Oxford, United Kingdom	Lead of Sub-projects; Study design	
Erik	von Elm		MD, MSc	Cochrane Germany Foundation	Freiburg, Germany	Collaborator; Project design of ASPIRE and other sub-projects	
Matthias	Briel		Prof	University Hospital Basel	Basel, Switzerland	Overall project lead	
