# Supplementary material for: Nonregistration, Discontinuation, and Nonpublication of Randomized Trials: A Systematic Review
Source: JAMA Netw Open. 2025 Sep 3;8(9):e2524440. doi: 10.1001/jamanetworkopen.2025.24440 (PMC12409572; doi:10.1001/jamanetworkopen.2025.24440)
Supplement: Supplement 3. — Data Sharing Statement [file jamanetwopen-e2524440-s003.pdf]

# Data Sharing Statement

Speich. Nonregistration, Discontinuation, and Nonpublication of Randomized Trials. *JAMA Netw Open*. Published September 03, 2025. doi:10.1001/jamanetworkopen.2025.24440

**Data**
**Data available:** No

**Additional Information**
**Explanation for why data not available:** All participating ethics committees were project partners and granted us access to confidential study protocols under the condition that only aggregated data will be made publicly available. All investigators signed a corresponding confidentiality agreement with ethics committees. All aggregated data that were used for this study are presented in Tables and Figures of the manuscript or the appendix.
